# Lateral Orbitofrontal Cortex Involvement in Initial Negative Aesthetic Impression Formation

**DOI:** 10.1371/journal.pone.0038152

**Published:** 2012-06-04

**Authors:** Enric Munar, Marcos Nadal, Jaume Rosselló, Albert Flexas, Stephan Moratti, Fernando Maestú, Gisèle Marty, Camilo J. Cela-Conde

**Affiliations:** 1 Human Evolution and Cognition Group (IFISC-CSIC), University of the Balearic Islands, Palma de Mallorca, Spain; 2 Cognitive and Computational Neuroscience Laboratory, Center for Biomedical Technology, Universidad Complutense & Politécnica de Madrid, Madrid, Spain; CSIC-Univ Miguel Hernandez, Spain

## Abstract

It is well established that aesthetic appreciation is related with activity in several different brain regions. The identification of the neural correlates of beauty or liking ratings has been the focus of most prior studies. Not much attention has been directed towards the fact that humans are surrounded by objects that lead them to experience aesthetic indifference or leave them with a negative aesthetic impression. Here we explore the neural substrate of such experiences. Given the neuroimaging techniques that have been used, little is known about the temporal features of such brain activity. By means of magnetoencephalography we registered the moment at which brain activity differed while participants viewed images they considered to be beautiful or not. Results show that the first differential activity appears between 300 and 400 ms after stimulus onset. During this period activity in right lateral orbitofrontal cortex (lOFC) was greater while participants rated visual stimuli as not beautiful than when they rated them as beautiful. We argue that this activity is associated with an initial negative aesthetic impression formation, driven by the relative hedonic value of stimuli regarded as not beautiful. Additionally, our results contribute to the understanding of the nature of the functional roles of the lOFC.

## Introduction

Human beings spontaneously assess the beauty –and lack of it– of many things in the world around them, including other humans, sceneries, objects, and artworks. Neuropsychological and neuroimaging studies have begun disclosing the neural underpinnings of this seemingly distinctive human trait [1]–[5]. Specifically, they have found positive aesthetic appreciation to be related with an enhancement of low-level sensory and high-level top-down processing, activation of cortical areas involved in evaluative judgment, and an engagement of the reward circuit, including cortical regions (insula, anterior cingulate, orbitofrontal and ventromedial prefrontal) and subcortical regions (caudate nucleus, susbtantia nigra, and nucleus accumbens), as well as some of the regulators of this circuit (amygdala, thalamus, hippocampus) [6].

Psychological models of visual aesthetics consider that viewers engage an artwork or design in two stages [7]. First, as soon as 300 ms after stimulus onset [8], the overall arrangement and meaning of the composition is extracted in the form of an initial impression. Second, after 600 ms specific features are scrutinized and the display is subjected to deeper aesthetic evaluation. It is then that the aesthetic experience proper arises. Given that most neuroimaging studies of aesthetic appreciation have used fMRI, their results most probably reflect processes underlying the second stage of visual aesthetic appreciation, while little is known of brain mechanisms involved in the initial impression formation.

Additionally, the neural substrate of neutral and negative aesthetic appreciation, i.e., the experience of aesthetic indifference towards an object, or a negative aesthetic impression of it, has received little attention. Most of the studies carried out to date have usually found that stimuli rated as ugly or as least preferred are associated with less activity in the same regions that are associated with positively rated stimuli. We believe that this could be a result of the stimuli sets used in neuroimaging studies, which typically do not include examples of images or sounds that would generally be considered to be truly not beautiful.

**Figure 1:**
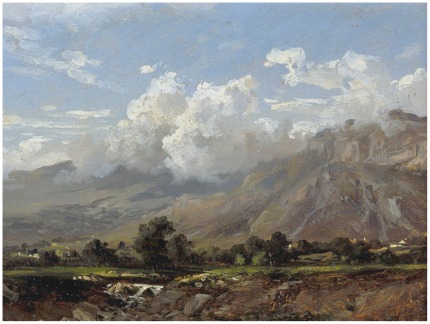
*Mountain Landscape* by Carlos de Haes. Reproduced with kind permission of Museo del Prado, Madrid (Spain).

**Figure 2:**
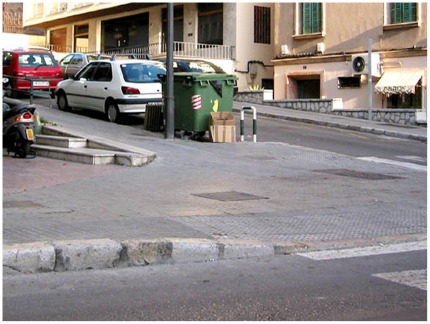
Photograph of an urban scene.

In the present study we assembled a set of 400 images including a broad range of artworks and photographs (see Figure 1 and Figure 2 for examples). The participants’ task was to decide whether each stimulus was beautiful or not. Half of the participants were asked to indicate the images that they thought were beautiful, while the other half were asked to do so for the images that they thought were not beautiful. Participants performed this task while their brain activity was being scanned by magnetoencephalography (MEG), affording a precise measurement of cortical activity and its time course for the first second after stimulus onset, ideal for analysis of the initial impression formation stage of aesthetic appreciation.

## Methods

### Participants

Ten female and 10 male university students (average age 24.45 years) at the Universidad Complutense (Madrid), with no previous training or special experience in art, volunteered to participate in this study. They all had normal or corrected vision and normal color vision. All were right-handed. All participants gave written informed consent. The experiment was approved by the Ethical Committee of the Comunitat Autònoma de les Illes Balears (Spain).

### Stimuli

All participants were presented with the same set of photographs of either artistic paintings or natural objects, belonging to 5 classes: (*i*) 50 pictures of abstract art; (*ii*) 50 pictures of classic art (Figure 1); (*iii*) 50 pictures of Impressionist art; (*iv*) 50 pictures of Postimpressionist art; (*v*) 200 photographs of landscapes, artifacts, urban scenes (Figure 2), and the like (true-life pictures from the Master Clips Premium Image Collection, IMSI, San Rafael, CA; the book *Boring Postcards*, London, Phaidon Press; and photographs taken by us). The artistic styles were decided following the collection *Movements in Modern Art* from the Tate Gallery, London, and we added European paintings of the XVII and XVIII centuries and Popular Art pictures. The objective was to present participants with a variety of artistic styles that could cover a broad range of aesthetic experiences. To avoid the activation of facial-recognition brain mechanisms, pictures containing close views of humans were not included. Four stimuli (2 artistic and 2 natural) were used for the participants’ preliminary training.

All stimuli were adjusted to the same resolution (150 pixels per inch) and dimensions (12×9 cm). They were homogenized by 3 operations. First, a semantic judgment test was performed to assess the effect of pictorial complexity on aesthetic appreciation [9]–[11]. A total of 711 stimuli were shown to 114 voluntary participants (undergraduate university students) on the screen of a computer. They were asked to rate each picture’s complexity from 1 to 10. All pictures receiving a mean <4.51 points were discarded. Second, the color spectrum of the visual stimuli was adjusted. We analyzed the 503 stimuli selected in the previous step, measuring their color spectrum by means of Adobe Photoshop 6.0 (Adobe Systems). The screen was calibrated with 9,300 white dot adjustment. Values of extreme illumination and shadow in each picture were adjusted to reach a global tone range allowing the best detail. Stimuli were classified according to their dominant tone (dark, medium, or light), and those with a mean distribution of pixels concentrated in both the left (dark) and right (light) extremes of the histogram were discarded. Third, the light reflected by stimuli (luminous emittance) was measured in a dark room; by means of a Minolta Auto Meter IV F digital photometer placed 40 cm from the screen with an accessory to 40° reflected light. Stimuli over 395 lux or below 365 lux were discarded. A total of 400 stimuli reasonably homogenized with regards to pictorial complexity, color spectrum, luminosity, and light reflection were thus obtained. Also, four stimuli (two artistic and two natural) were selected for the participants’ training tasks. Four additional stimuli (two artistic and two natural) were projected as initial pictures in the MEG experiment, their results being discarded to avoid the primacy effect.

### Procedure

We used a two-alternative forced choice (2AFC) design, with two response levels: (a) beautiful, and (b) not beautiful. Half of the male and half of the female participants were asked to indicate, by raising the index finger-detected by a specific device fixed to the finger-, that they found the image to be beautiful, and the rest of the images were considered as not beautiful. The other half of the participants were asked to raise a finger if they thought the image was not beautiful and, thus, the remaining images were considered as beautiful. The session consisted of 4 images for practice and 400 images as trials. Every image was presented for 3 seconds, followed by an interstimulus interval (black screen) of between 1900 and 2100 ms. Image presentation order was randomized. The technique used to register brain activity was MEG. Before going into the MEG isolated room, participants received a short briefing about the technique and the aesthetic appreciation task they were required to carry out.

During the MEG recording, the stimuli were generated by a computer running the SuperLab application. The pictures were projected through an LCD video projector situated outside of the shielded room onto a series of mirrors located inside, the last of which–the one where the stimuli were shown to the participants– was suspended ≈1 m above the subject’s face. The pictures subtended 1.8° and 3° vertical and horizontal visual angle, respectively.

After the MEG session, each participant performed a behavioral test. They were asked to rate the beauty of each of the same stimuli that they saw previously on a 1 to 9 Likert scale.

### Image Acquisition

The methods underlying MEG data collection and analysis are described in [12] and are outlined only briefly here. MEG recordings were made with a whole-head neuromagnetometer (Magnes 2500 WH, 4-D Neuroimaging) consisting of 148 magnetometer coils. The instrument is housed in a magnetically shielded room designed to reduce environmental magnetic noise that might interfere with biological signals. The variables taken into account in the MEG protocol and the procedure were the following:

- Signal analysis. The MEG signal was filtered “online” with a bandpass filter set between.1 and 50 Hz, and digitalized with a sampling rate of 254 Hz, during a time window of 1,050 ms including a 150 ms prestimulus period. The epoch data obtained for each participant were baseline-corrected and noise-reduced. Each single trial event-related field was visually assessed to reject those exhibiting eye movements, blinks, or movement artifacts. Artifact-free epochs of each channel and participant were averaged across each condition. The minimum number of trials obtained after artifact rejection was 80.
- Source analyses. The MNE procedure, commonly used in MEG source reconstruction and described in detail elsewhere [13], was used for estimating the cortical origin of the brain response. Because MEG sources are believed to be restricted to the pyramidal neurons of the cortex [14], the dipoles of the source space model were restricted to a cortical surface extracted from a structural MRI. A tessellated cortical mesh template surface derived from the Montreal Neurological Institute (MNI) phantom brain [15] and implemented in SPM (www.fil.ion.ucl.ac.uk/spm) served as a brain model to estimate the current source distribution. Typically the dipoles of the distributed source model are evenly placed at each node of the mesh representing the white/gray matter interface [16]. The SPM template used contained 3,004 dipole locations. This dipole mesh was used to calculate the forward solution using a spherical head model. A spherical head model is known to be sufficient to estimate a good approximation of the physical head properties and to compute the magnetic field propagation of the forward model [17]. The inverse solution (the estimation of the current source density based on the MEG topography) was calculated using the l2 Minimum Norm solution implemented in “in-house MatLab-code.” To estimate the underlying current source density (the source strength at each node of the MNI phantom brain) of the evoked field, the MNE was computed for each time point, participant, and condition. Finally, for each participant and condition, the MNE solutions were divided in 100-ms steps and averaged across the time windows. The resulting MNE averages were submitted to Statistical Parametric Mapping (SPM) analysis.

## Results

**Figure 3:**
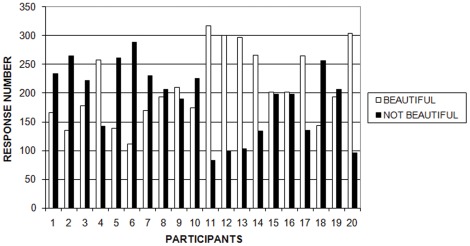
Number of Responses (beautiful and not beautiful) for each participant.

First, we examined the number of responses provided by each subject. As shown in Figure 3, there were important individual differences in the number of responses given in each condition: beautiful and not beautiful. However, there were no significant differences between the number of beautiful and not-beautiful responses taken the group as a whole [t(19) = .67, *p* = .43]. Ten participants gave more “beautiful” than “not beautiful” answers and the other ten participants gave more “not beautiful” than “beautiful” answers. The lowest number of responses were 111 and 83 for beautiful and not beautiful, respectively. These values fulfill the minimum value of 80 that we set to have enough stimuli for a suitable analysis.

**Figure 4:**
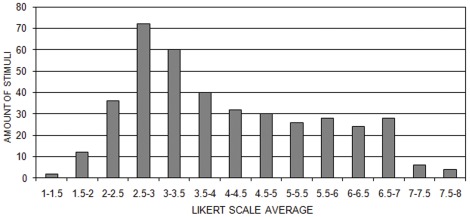
Amount of Stimuli according Likert Scale Average intervals in all participants.

Although the number of “beautiful” and “not beautiful” answers were quite balanced in the 2AFC task, the results of the behavioral test using the Likert scale on the beauty showed a general trend toward a negative assessment (Figure 4). The general mean was 4.11. It was significantly lower than the expected value (5) in a scale from 1 to 9.

In light of previous results [18], we analyzed the possible differences between sexes. We found no differences in the frequencies of beautiful and not-beautiful judgments between both sexes [χ^2^(1) = 0.0023, *p* = 0.96]. We assayed the differences between men and women’s ratings of the 400 stimuli by means of Student’s *t* tests. There were no significant differences between men and women’s beauty ratings for any of the 400 stimuli.

The images were analyzed by means of the SPM8 (Statistical Parametric Mapping) software, implemented on MatLab 7.9, using the module M/EEG. The experimental design included a within-groups variable (aesthetic preference, with the levels beautiful and not beautiful). Differences between the levels were contrasted by means of *t* tests (implemented on SPM8) with a *p*<.001 (*t* = 3.332624) with no adjustment to control. The extent threshold was set to *k* = 10 voxels. The images were divided in 100-ms steps and averaged across the time windows, as noted in the method section. Thus, we obtained a brain activity difference map for each 100 ms, from 0 to 900 after stimulus onset (see Supporting Results S1). The statistically significantly differences found correspond to brain activity in participants when comparing not-beautiful and beautiful stimuli.

**Figure 5:**
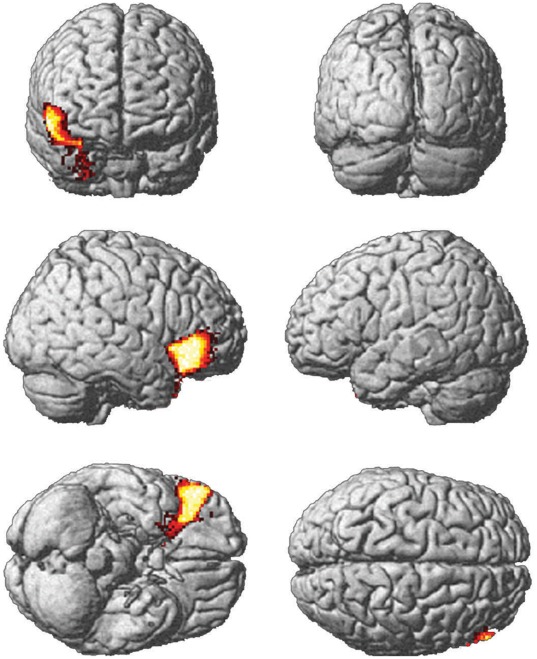
Activity significantly greater for stimuli rated as not beautiful rather than beautiful: 300–400 ms time-window.

Our results indicate that whereas images rated as beautiful were associated with activity in parietal regions from 300 ms onward [18], images rated as not beautiful were associated with activity in the right lateral orbitofrontal cortex between only 300 and 400 ms after stimulus onset (Figure 5 and Supporting Results S1) with a peak at 58/42/10 MNI coordinates. Hence, brain activity associated with images which participants rated as not beautiful was clearly distinct to that associated with images rated as beautiful. There were no brain activity differences between men and women at any time when rating not beautiful images.

## Discussion

The results of this study and that performed by [18] have shown that differences in brain activity related with beautiful and not beautiful images appear between 300 and 400 ms after stimuli presentation. Whereas beautiful images are related with activity in parietal regions [18], not beautiful visual stimuli are related with activity in right lateral orbitofrontal cortex (OFC) at a specific point in time (300–400 ms).

Our results coincide with the temporal data obtained by EEG in Jacobsen and Höfel’s aforementioned studies [8], [19]. There, participants judged geometric graphic patterns using yes/no responses for symmetry and beauty while electrophysiological activity was recorded. At frontal sites, a more negative-going phasic deflection was observed for the not beautiful judgments from about 300 to 400 ms after stimulus onset. Temporal and spatial aspects of their results were relatively similar to ours. However, the authors suggested that that activity was generated in the anterior frontomedian cortex according to other fMRI brain imaging studies in evaluative judgment [20], though they did note that their ERP topography was also consistent with other, less likely, generator configurations. Our results show that the right lateral OFC was the activity source of brain activity related with non-beautiful stimuli. It seems, overall, that both results share commonalities, especially in relation to their coincidence in time and the frontal origin. The differences could owe mainly to the kind of stimuli-geometrical designs in [8]-and the task procedure.

Several fMRI studies of aesthetic appreciation have identified activity in the OFC. This has been interpreted as a reflection of affective processes involved in aesthetic appreciation, given the well established role of the OFC in coding stimulus reward value, forging associations between diverse stimuli and primary reinforcers, and the prediction of future reward [21]. Such activity, however, appeared while participants viewed images they rated as beautiful or liked a lot [4], [22], [23], and was located in a much more medial region of the OFC. Our results, which support Kirk’s [23] finding that activity in the right lateral OFC correlated negatively with aesthetic ratings, suggest that the role of the OFC in aesthetic preference is not limited to the representation of the reward value of stimuli regarded as beautiful. This region, especially its lateral aspect, also seems to play a role in the processing of stimuli rated as not beautiful. The fact that this activity is unilaterally right-sided as opposed to bilateral is in agreement with observations that such functional asymmetry is common in tasks involving no complex linguistic processes [24].

A number of neuroimaging studies have suggested that lateral OFC and medial OFC have different functional roles [23], [25]. The lateral OFC seems to be especially involved in the evaluation of punishers, whereas the medial OFC seems to be especially involved in the monitorization of reinforcer reward value [21], [26], [27], [28]. However, some authors cautioned against the straightforward dissociation of lateral and medial OFC in terms of valence [29]. Under this perspective, while medial OFC monitors current reinforcement relations, especially in familiar or constant situations, the lateral regions of the OFC, in particular the caudal one, are involved in the inhibition of previously rewarded responses during an ongoing task, especially when the situation is variable or uncertain. Thus, rather than representing different values, medial and lateral regions are thought to perform different functions in relation to reward [30]. Specifically, the medial OFC would be involved in establishing associations between stimuli and rewarded responses. Activity in the lateral OFC, on the other hand, would reflect dealing with uncertainty, and suppressing responses that had previously been rewarded. However, given that there might be no clear way of separating negative affect from the experience of uncertainty and conflict related with behavioral shifting and the conflict between approach and withdrawal responses [24], it has been suggested that activity in the lateral OFC might be related with feelings of uncertainty and the need to prepare new responses or change behavior when negatively valued stimuli are presented [24], [31].

An important issue refers to the recent demonstration that OFC neurons encode relative rather than absolute reward preference or relative pleasantness in humans [31], [32]. As already demonstrated in primates [33], the medial and lateral OFC respond to the same perceptual stimulus depending on the relative value context and not on the absolute value of the reward. When the stimulus B represented the more valuable reward of two alternatives (AB), the medial OFC enhanced its activity. And when the same stimulus B represented the less valuable reward from another comparison (BC), the lateral rOFC enhanced its activity. Thus, OFC seems to encode relative rather than absolute reward value. This suggests that the activity of the rOFC identified in the present work could be associated with the relative unpleasantness of non-beautiful images –whether they were encoded as aesthetically neutral or as aesthetically negative images in absolute terms– as being compared with the beautiful ones. This way, the results obtained would be due to the context-dependent effect induced by the 2AFC task. In accordance to this interpretation, recent studies argue that the OFC plays a central role in value comparison, and that the critical weighting is based on the differences between options [34]. So, in a certain way, the “not beautiful” option could be negatively biased by the comparison with the beautiful one.

We thus propose that activity identified in the present study in the right lateral OFC corresponds essentially to an early representation of the relative negative value of stimuli rated as not beautiful. How is this initial negative aesthetic impression formation related with subsequent stages? Given the high temporal resolution of MEG, the results of this and previous studies reveal some facts about the time course of neural activity underlying the aesthetic experience. First, brain activity related specifically with the aesthetic quality of visual stimuli begins between 300 and 400 ms after stimulus onset. This is true both for stimuli regarded as beautiful, as shown in a related article [18], and those regarded as not beautiful, as shown in the present study. This suggests that neural processes specifically underlying aesthetic appreciation occur after the initial perceptual processes have been completed. Second, judgments of stimuli as beautiful or not beautiful are associated with different processes in different brain regions. Whereas beautiful images are more related than non beautiful ones with parietal activity, presumably involved in the analysis of the stimuli’s spatial features [18], images considered as not beautiful are more strongly associated than beautiful ones with activity in the lateral orbitofrontal cortex, underlying the representation of their negative reward value, as argued here. Third, the duration of processes specifically involved in rating stimuli as beautiful and not beautiful seems to be different. Whereas activity related with beautiful stimuli lasted at least over half a second, from 300 to 900 ms after stimulus onset [18], activity specifically related with stimuli rated as not beautiful was only detected in the 300 to 400 ms window. This, however, does not mean that these stimuli were not processed any further. It means that subsequent processing was not particular to non beautiful stimuli.

These results, nevertheless, must not be taken as a demonstration that there are specific brain regions for aesthetic processing, or even for processing specific aesthetic qualities, such as beauty or ugliness. Although we have presented the results of a local analysis of neural activity, registered using MEG, brain function can clearly be analyzed at different scales. These scales can be more local, like the study presented here, or more global, like our time-frequency analysis, which revealed that beauty appreciation was associated with a greater oscillatory power of four frequency bands [5]. Such a mechanism has the potential to explain the coordinated interaction of processes in different brain regions whose activity seems to be contingent with aesthetic appreciation [6], [35]. A truly comprehensive understanding of the neural processes underlying aesthetic experience will require both kinds of studies, and a better understanding of the relation between local and global brain processes.

## Supporting Information

Supporting Results S1 **Analysis Results from SPM8 (Statistical Parametric Mapping).**
(PDF)
