# Supplementary material for: Lateral Orbitofrontal Cortex Involvement in Initial Negative Aesthetic Impression Formation
Source: PLoS One. 2012 Jun 4;7(6):e38152. doi: 10.1371/journal.pone.0038152 (PMC3367021; doi:10.1371/journal.pone.0038152)
Supplement: Supporting Results S1 — Analysis Results from SPM8 (Statistical Parametric Mapping). (PDF) [file pone.0038152.s001.pdf]

Supplementary Results

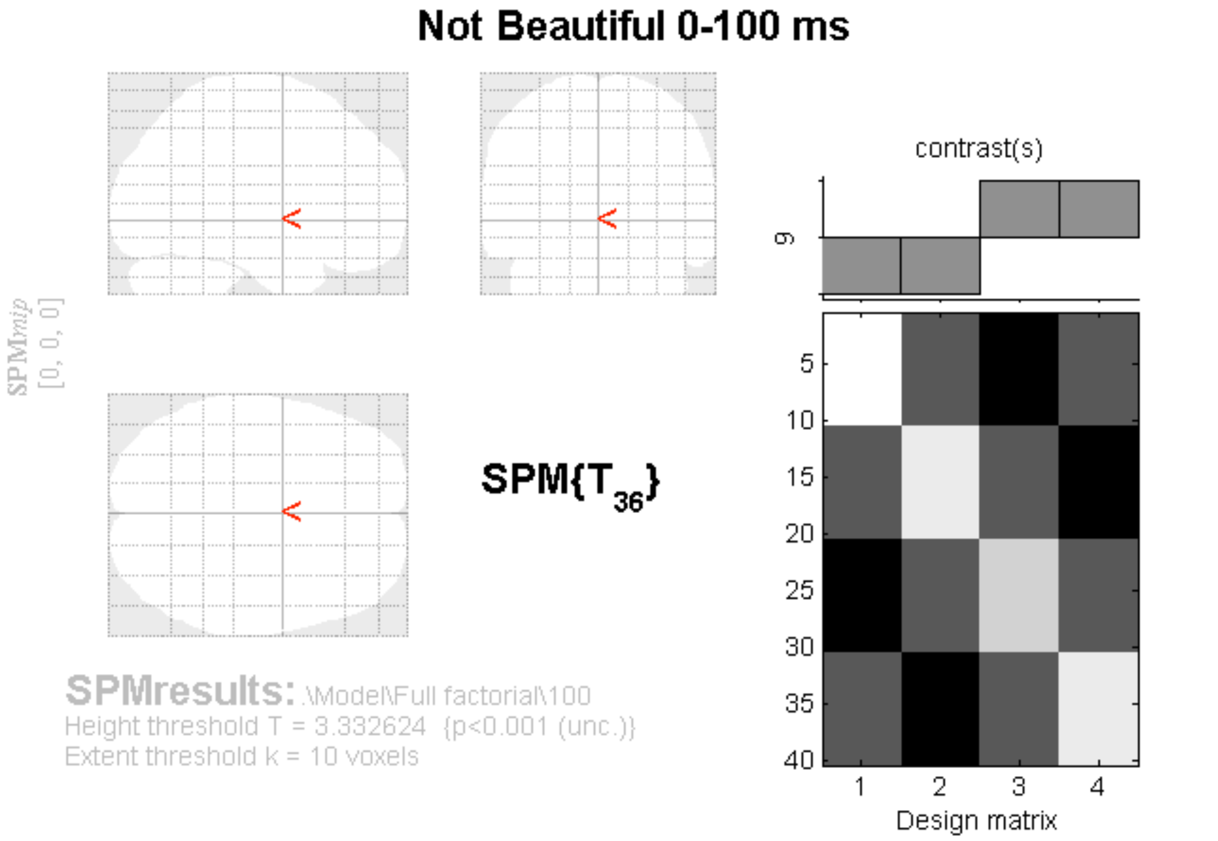

Statistics: ***p-values adjusted for search volume***

set-level		cluster-level				peak-level					mm mm mm
p	c	$p_{FWE-corr}$	$q_{FDR-corr}$	k_E	p_{uncorr}	$p_{FWE-corr}$	$q_{FDR-corr}$	T	$(Z_{\equiv})$	p_{uncorr}	

no suprathreshold clusters

table shows 3 local maxima more than 8.0mm apart

Height threshold: T = 3.33, p = 0.001 (0.999)
Extent threshold: k = 10 voxels, p = 0.641 (0.988)
Expected voxels per cluster, <k> = 44.712
Expected number of clusters, <c> = 4.46
FWEp: 5.464, FDRp: Inf, FWEc: Inf, FDRc: Inf

Degrees of freedom = [1.0, 36.0]
FWHM = 15.2 15.9 15.3 mm mm mm; 7.6 7.9 7.7 {voxels}
Volume: 2093352 = 261669 voxels = 529.8 resels
Voxel size: 2.0 2.0 2.0 mm mm mm; (resel = 463.36 voxels)

Not Beautiful 100-200 ms

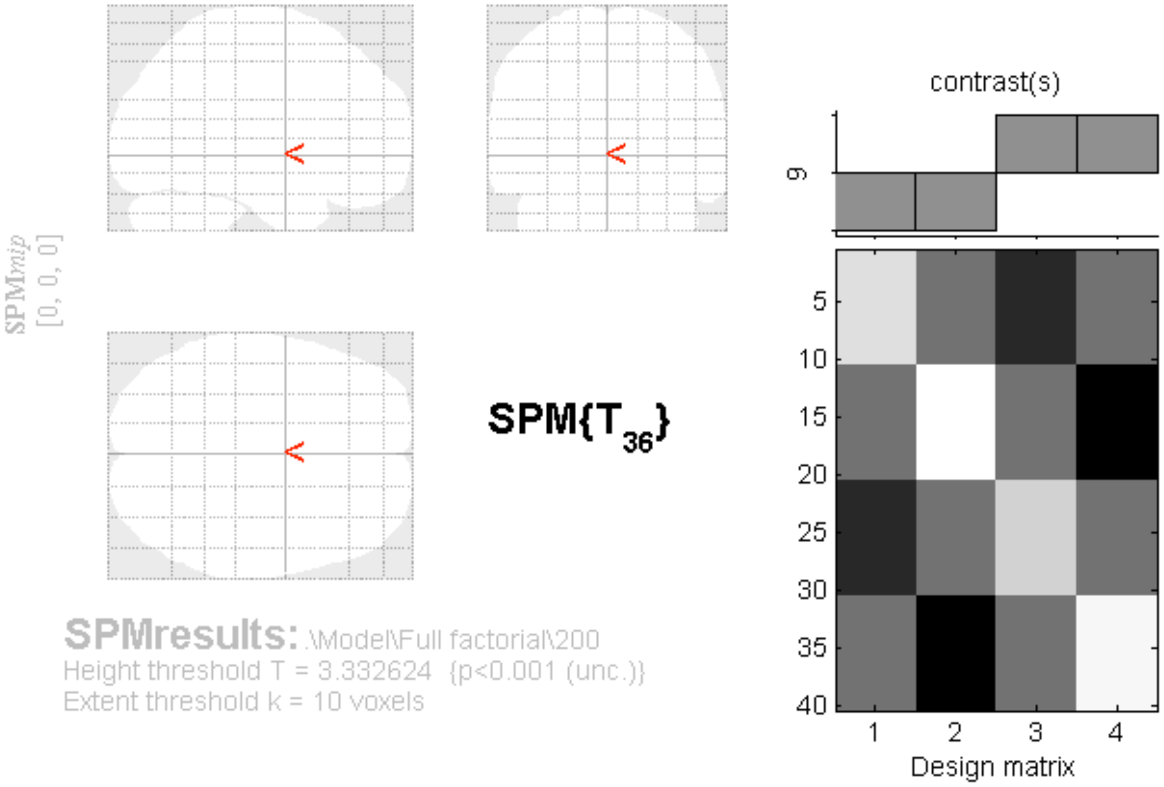

Statistics: ***p-values adjusted for search volume***

set-level		cluster-level				peak-level					mm mm mm
p	*c*	$p_{FWE\text{-}corr}$	$q_{FDR\text{-}corr}$	k_E	p_{uncorr}	$p_{FWE\text{-}corr}$	$q_{FDR\text{-}corr}$	*T*	$(Z_{\equiv})$	p_{uncorr}	

no suprathreshold clusters

table shows 3 local maxima more than 8.0mm apart

Height threshold: T = 3.33, p = 0.001 (0.998)
Extent threshold: k = 10 voxels, p = 0.665 (0.984)
Expected voxels per cluster, <k> = 50.877
Expected number of clusters, <c> = 4.11
FWEp: 5.416, FDRp: Inf, FWEc: Inf, FDRc: Inf

Degrees of freedom = [1.0, 36.0]
FWHM = 16.1 16.2 16.2 mm mm mm; 8.0 8.1 8.1 {voxels}
Volume: 2093424 = 261678 voxels = 465.5 resels
Voxel size: 2.0 2.0 2.0 mm mm mm; (resel = 527.26 voxels)

Not Beautiful 200-300 ms

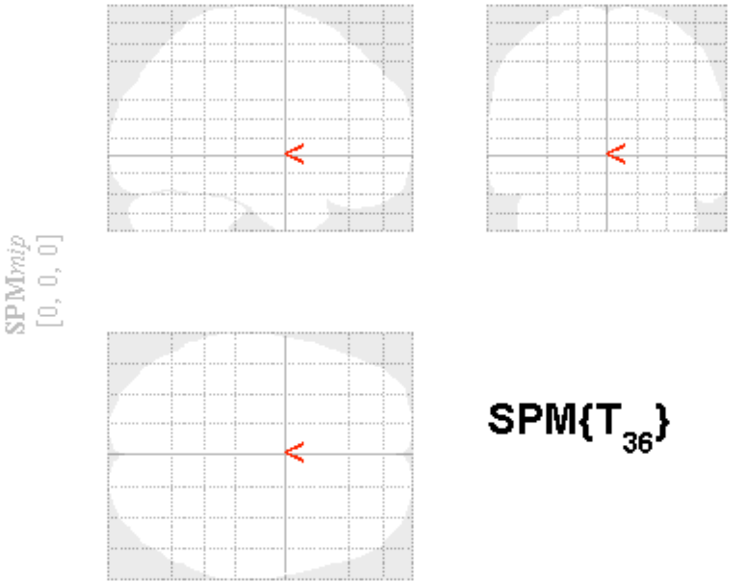

SPM{T_{36}}

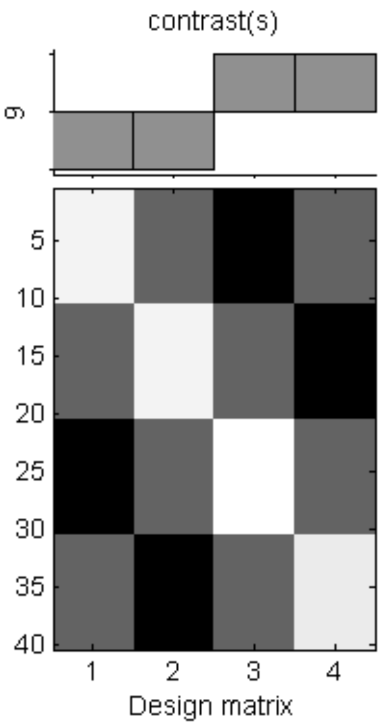

SPMresults: .\Model\Full factorial\300
Height threshold T = 3.332624 {p<0.001 (unc.)}
Extent threshold k = 10 voxels

Statistics: ***p-values adjusted for search volume***

set-level		cluster-level				peak-level					mm mm mm
p	c	$p_{FWE-corr}$	$q_{FDR-corr}$	k_E	p_{uncorr}	$p_{FWE-corr}$	$q_{FDR-corr}$	T	($Z_{\equiv}$)	p_{uncorr}	

no suprathreshold clusters

table shows 3 local maxima more than 8.0mm apart

Height threshold: T = 3.33, p = 0.001 (0.885)
Extent threshold: k = 10 voxels, p = 0.827 (0.833)
Expected voxels per cluster, <k> = 160.302
Expected number of clusters, <c> = 1.79
FWEp: 4.986, FDRp: Inf, FWEc: Inf, FDRc: Inf

Degrees of freedom = [1.0, 36.0]
FWHM = 23.7 23.9 23.5 mm mm mm; 11.8 12.0 11.7 {voxels}
Volume: 2078256 = 259782 voxels = 146.6 resels
Voxel size: 2.0 2.0 2.0 mm mm mm; (resel = 1661.25 voxels)

Not Beautiful 300-400 ms

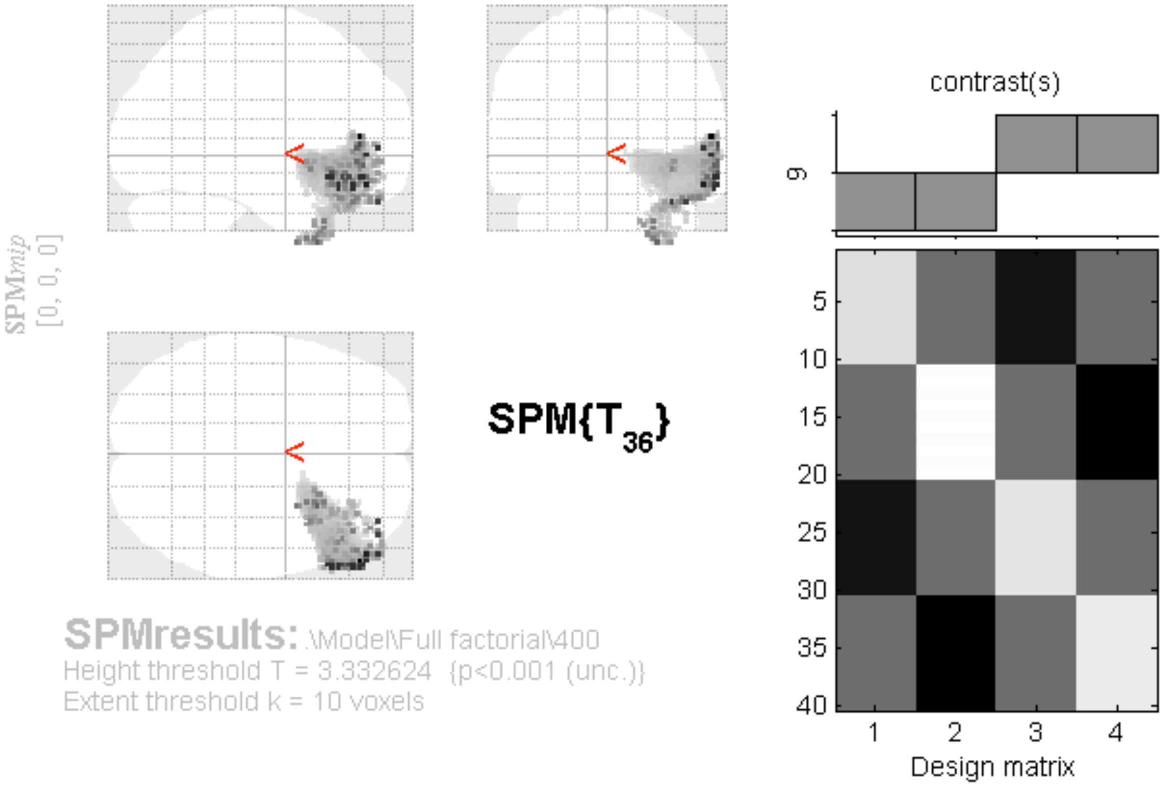

Statistics: ***p-values adjusted for search volume***

set-level		cluster-level				peak-level					mm mm mm		
p	c	$p_{FWE-corr}$	$q_{FDR-corr}$	k_E	p_{uncorr}	$p_{FWE-corr}$	$q_{FDR-corr}$	T	$(Z_{\equiv})$	p_{uncorr}			
0.770	2	0.000	0.000	2913	0.000	0.119	0.836	4.85	4.23	0.000	58	42	10
						0.195	0.836	4.63	4.07	0.000	54	46	-16
						0.218	0.836	4.57	4.03	0.000	58	46	0
		0.893	0.941	24	0.601	0.282	0.836	4.45	3.95	0.000	36	52	-26
						0.736	0.836	3.84	3.49	0.000	48	54	-24

table shows 3 local maxima more than 8.0mm apart

Height threshold: T = 3.33, p = 0.001 (0.976)
Extent threshold: k = 10 voxels, p = 0.753 (0.939)
Expected voxels per cluster, <k> = 87.697
Expected number of clusters, <c> = 2.80
FWEp: 5.210, FDRp: Inf, FWEc: 2913, FDRc: 2913

Degrees of freedom = [1.0, 36.0]
FWHM = 19.1 19.8 19.3 mm mm mm; 9.6 9.9 9.6 {voxels}
Volume: 2076168 = 259521 voxels = 267.8 resels
Voxel size: 2.0 2.0 2.0 mm mm mm; (resel = 908.83 voxels)

Not Beautiful 400-500 ms

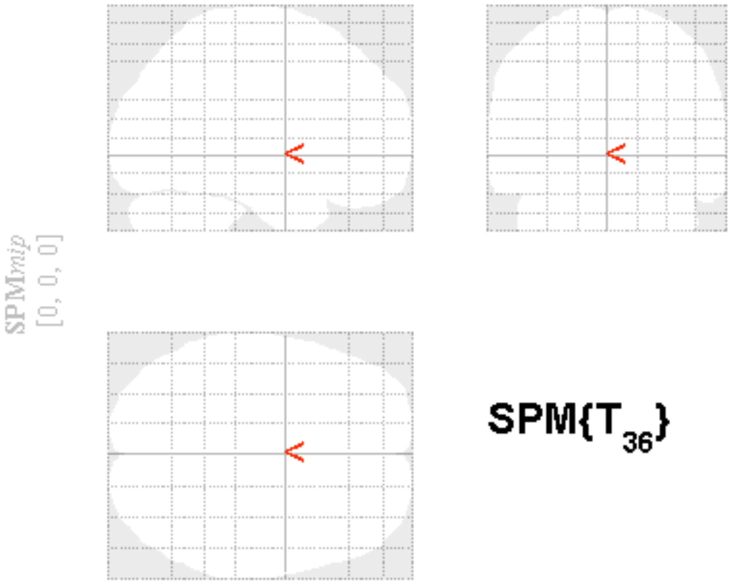

SPM{T_{36}}

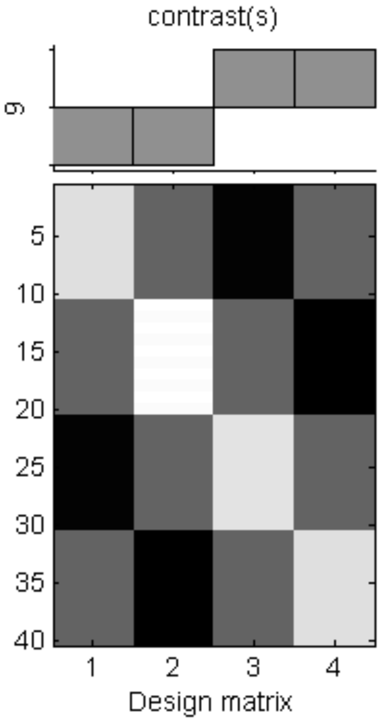

SPMresults: .\Model\Full factorial\500
Height threshold T = 3.332624 {p<0.001 (unc.)}
Extent threshold k = 10 voxels

Statistics: ***p-values adjusted for search volume***

set-level		cluster-level				peak-level					mm mm mm
p	*c*	$p_{FWE\text{-}corr}$	$q_{FDR\text{-}corr}$	k_E	p_{uncorr}	$p_{FWE\text{-}corr}$	$q_{FDR\text{-}corr}$	*T*	($Z_{\equiv}$)	p_{uncorr}	

no suprathreshold clusters

table shows 3 local maxima more than 8.0mm apart

Height threshold: T = 3.33, p = 0.001 (0.837)
Extent threshold: k = 10 voxels, p = 0.846 (0.784)
Expected voxels per cluster, <k> = 194.043
Expected number of clusters, <c> = 1.53
FWEp: 4.911, FDRp: Inf, FWEc: Inf, FDRc: Inf

Degrees of freedom = [1.0, 36.0]
FWHM = 25.4 25.8 24.5 mm mm mm; 12.7 12.9 12.3 {voxels}
Volume: 2056960 = 257120 voxels = 119.9 resels
Voxel size: 2.0 2.0 2.0 mm mm mm; (resel = 2010.93 voxels)

Not Beautiful 500-600 ms

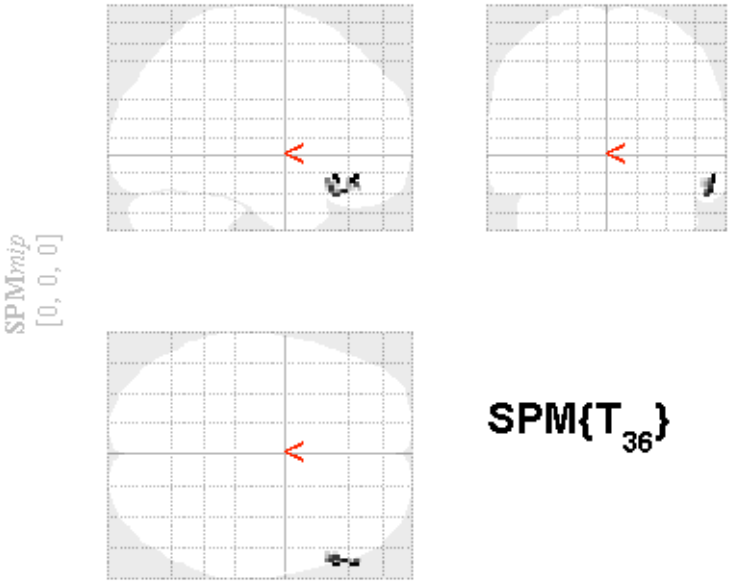

SPM{T_{36}}

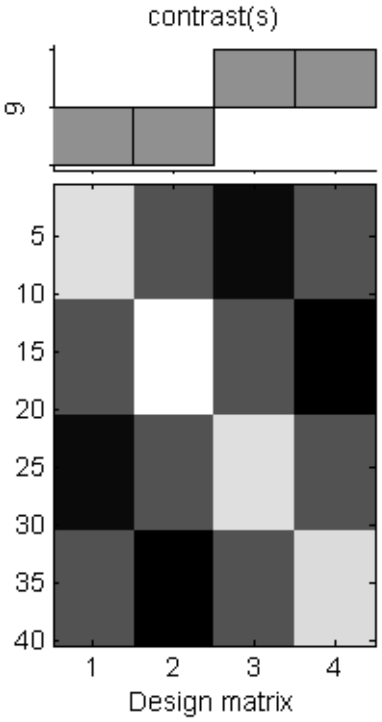

SPMresults: .\Model\Full factorial\600
Height threshold T = 3.332624 {p<0.001 (unc.)}
Extent threshold k = 10 voxels

Statistics: *p-values adjusted for search volume*

set-level	cluster-level				peak-level					mm mm mm
	$p_{FWE-corr}$	$q_{FDR-corr}$	k_E	p_{uncorr}	$p_{FWE-corr}$	$q_{FDR-corr}$	T	$(Z_\equiv)$	p_{uncorr}	
	0.583	0.975	30	0.785	0.372	0.805	3.76	3.43	0.000	60 30 -14
					0.372	0.805	3.76	3.43	0.000	60 40 -12
					0.428	0.805	3.67	3.36	0.000	56 30 -22

table shows 3 local maxima more than 8.0mm apart

Height threshold: T = 3.33, p = 0.001 (0.672)
Extent threshold: k = 10 voxels, p = 0.890 (0.629)
Expected voxels per cluster, <k> = 335.884
Expected number of clusters, <c> = 0.99
FWEp: 4.707, FDRp: Inf, FWEc: Inf, FDRc: Inf

Degrees of freedom = [1.0, 36.0]
FWHM = 30.4 31.6 28.9 mm mm mm; 15.2 15.8 14.5 {voxels}
Volume: 2047464 = 255933 voxels = 68.9 resels
Voxel size: 2.0 2.0 2.0 mm mm mm; (resel = 3480.87 voxels)

Not Beautiful 600-700 ms

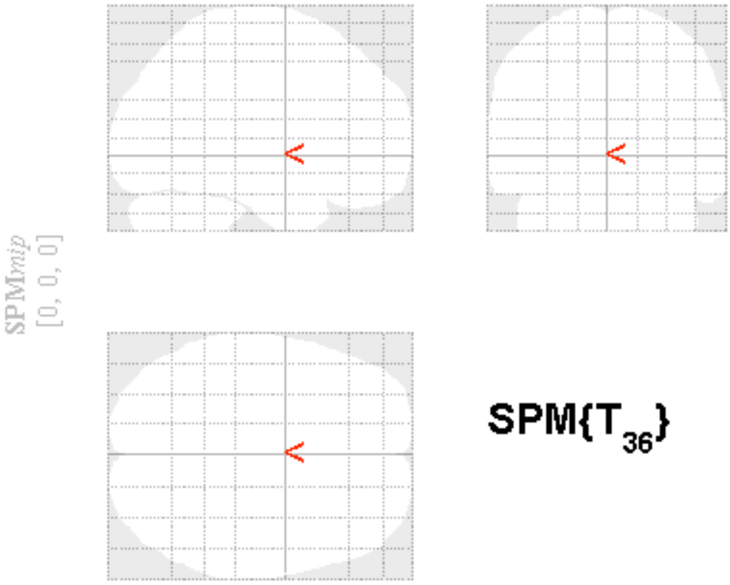

SPM{T_{36}}

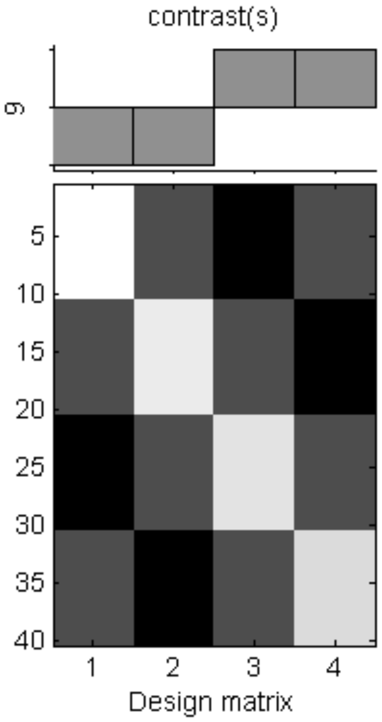

SPMresults: .\Model\Full factorial\700
Height threshold T = 3.332624 {p<0.001 (unc.)}
Extent threshold k = 0 voxels

Statistics: ***p-values adjusted for search volume***

set-level		cluster-level				peak-level					mm mm mm
p	*c*	$p_{FWE\text{-}corr}$	$q_{FDR\text{-}corr}$	k_E	p_{uncorr}	$p_{FWE\text{-}corr}$	$q_{FDR\text{-}corr}$	*T*	($Z_{\equiv}$)	p_{uncorr}	

no suprathreshold clusters

table shows 3 local maxima more than 8.0mm apart

Height threshold: T = 3.33, p = 0.001 (0.649)
Extent threshold: k = 0 voxels, p = 1.000 (0.649)
Expected voxels per cluster, <k> = 361.643
Expected number of clusters, <c> = 1.05
FWEp: 4.680, FDRp: Inf, FWEc: Inf, FDRc: Inf

Degrees of freedom = [1.0, 36.0]
FWHM = 31.2 32.1 30.0 mm mm mm; 15.6 16.0 15.0 {voxels}
Volume: 2052664 = 256583 voxels = 64.2 resels
Voxel size: 2.0 2.0 2.0 mm mm mm; (resel = 3747.82 voxels)

Not Beautiful 700-800 ms

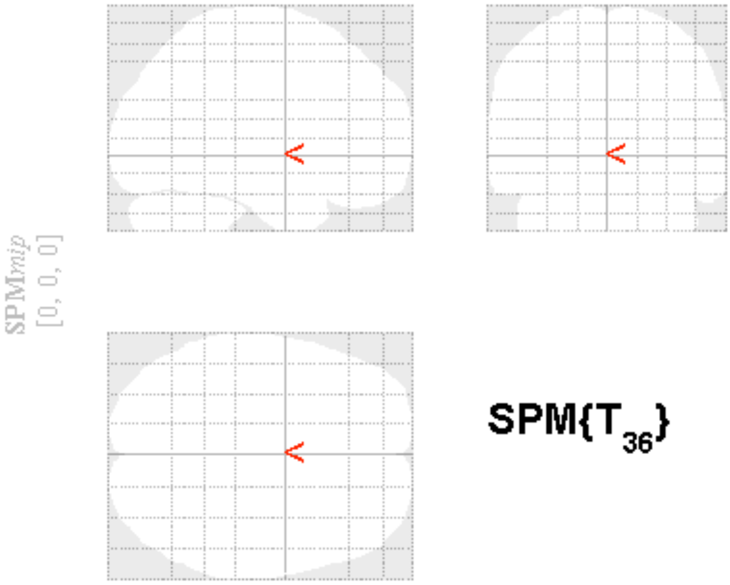

SPM{T_{36}}

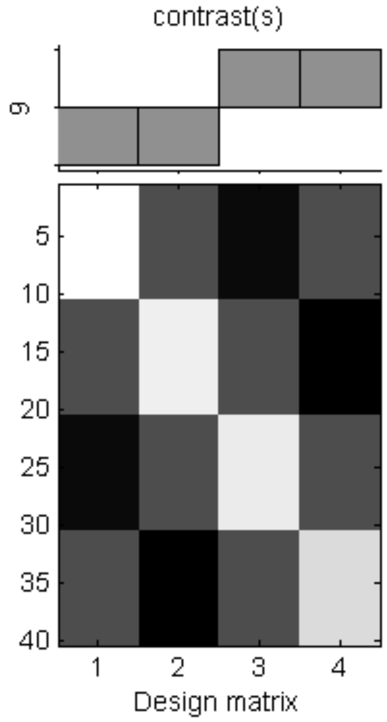

SPMresults: .\Model\Full factorial\800
Height threshold T = 3.332624 {p<0.001 (unc.)}
Extent threshold k = 0 voxels

Statistics: ***p-values adjusted for search volume***

set-level		cluster-level				peak-level					mm mm mm
p	*c*	$p_{FWE\text{-}corr}$	$q_{FDR\text{-}corr}$	k_E	p_{uncorr}	$p_{FWE\text{-}corr}$	$q_{FDR\text{-}corr}$	*T*	($Z_\equiv$)	p_{uncorr}	

no suprathreshold clusters

table shows 3 local maxima more than 8.0mm apart

Height threshold: T = 3.33, p = 0.001 (0.513)
Extent threshold: k = 0 voxels, p = 1.000 (0.513)
Expected voxels per cluster, <k> = 557.385
Expected number of clusters, <c> = 0.72
FWEp: 4.519, FDRp: Inf, FWEc: Inf, FDRc: Inf

Degrees of freedom = [1.0, 36.0]
FWHM = 35.6 37.5 34.6 mm mm mm; 17.8 18.7 17.3 {voxels}
Volume: 2040512 = 255064 voxels = 41.4 resels
Voxel size: 2.0 2.0 2.0 mm mm mm; (resel = 5776.34 voxels)

Not Beautiful 800-900 ms

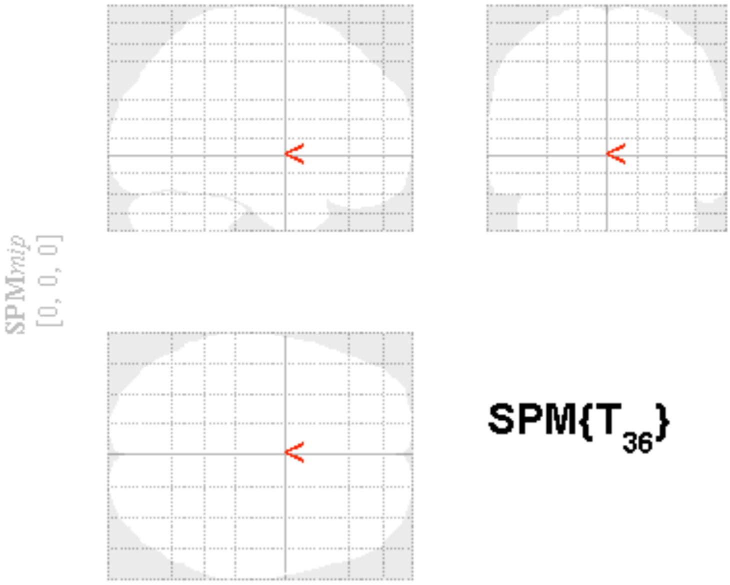

$SPM\{T_{36}\}$

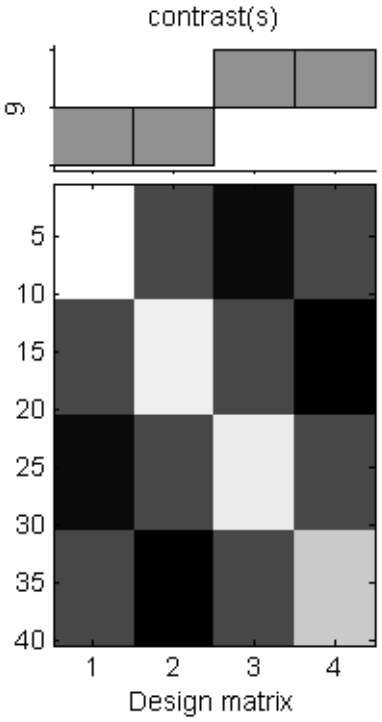

SPMresults: .\Model\Full factorial\900
Height threshold T = 3.332624 {p<0.001 (unc.)}
Extent threshold k = 0 voxels

Statistics: ***p-values adjusted for search volume***

set-level		cluster-level				peak-level					mm mm mm
p	c	$p_{FWE-corr}$	$q_{FDR-corr}$	k_E	p_{uncorr}	$p_{FWE-corr}$	$q_{FDR-corr}$	T	$(Z_{\equiv})$	p_{uncorr}	

no suprathreshold clusters

table shows 3 local maxima more than 8.0mm apart

Height threshold: T = 3.33, p = 0.001 (0.586)
Extent threshold: k = 0 voxels, p = 1.000 (0.586)
Expected voxels per cluster, <k> = 441.453
Expected number of clusters, <c> = 0.88
FWEp: 4.607, FDRp: Inf, FWEc: Inf, FDRc: Inf

Degrees of freedom = [1.0, 36.0]
FWHM = 33.3 34.1 32.3 mm mm mm; 16.6 17.1 16.1 {voxels}
Volume: 2053608 = 256701 voxels = 52.6 resels
Voxel size: 2.0 2.0 2.0 mm mm mm; (resel = 4574.91 voxels)
